# Enhancing corneal ectasia susceptibility detection: analysis of a new algorithm (BAD-D v4)

**DOI:** 10.1038/s41598-024-81809-w

**Published:** 2024-12-04

**Authors:** Bernardo T Lopes, Michael W Belin, Maria A. Henriquez, Luis Izquierdo, Thomas Kohnen, Renato Ambrosio

**Affiliations:** 1 Ophthalmology Department, Alder Hey Children’s NHS Foundation Trust, Liverpool, UK; 2 Ophthalmology Department, Federal Univeristy of São Paulo, São Paulo, Brazil; 3 Rio de Janeiro Corneal Tomography and Biomechanics Study Group, Rio de Janeiro, Brazil; 4 Department of Ophthalmology & Vision Science, University of Arizona, Tucson, AZ USA; 5 Research Department, Oftalmosalud Insituto de Ojos, Lima, Peru; 6 Universidad Ricardo Palma, Lima, Peru; 7 Universidad Nacional Mayor de San Marco, Lima, Peru; 8 Department of Ophthalmology, Goethe-University, Frankfurt am Main, Germany; 9 Federal Univerisity of the State of Rio de Janeiro (UNIRIO), Rio de Janeiro, Brazil

**Keywords:** Corneal diseases, Risk factors

## Abstract

Accurate detection of post-refractive ectasia susceptibility is essential during preoperative evaluation for laser vision correction (LVC) due to the risk of progressive corneal ectasia and vision decline post-surgery. Despite improved screening and a reduced incidence from 0.66 to 0.033%, iatrogenic ectasia remains a concern due to the severe vision loss it can cause, highlighting the need for more accurate detection tools. A new optimized version of the Belin/Ambrósio Enhanced Ectasia Display version 4 (BAD-D v4) was developed and validated across 26 international centers to enhance the detection of keratoconus and very asymmetric ectasia and to assess the risk of post-refractive ectasia. Analyzing a dataset of 3,886 eyes from 3,351 patients, including normal, keratoconus (KC), and cases with very asymmetric ectasia (VAE) categories, having one eye with normal topography (VAE-NT and the fellow eye with clinical ectasia (VAE-E). The study utilized an optimized logistic regression algorithm improving diagnostic accuracy. The BAD-D v4 showed superior efficacy in differentiating normal eyes from ectatic conditions, with Area Under the Receiver Operating Characteristic Curve (AUROC) scores of 0.997 and 0.998 in training and testing samples for normal versus clinical ectasia. Additionally, in Normal vs. Disease (KC + VAE), the AUROC was 0.974 and 0.966, and in the challenging Normal vs. VAE-NT diverse group, it scored 0.905 and 0.858. These results outperformed the current version (BAD-D v3) and were comparable to the Pentacam Random Forest Index in all tested scenarios, highlighting the potential of BAD-D v4 in early ectasia detection, without altering the index scale or the end-user experience.

**Supplementary Information:**

The online version contains supplementary material available at 10.1038/s41598-024-81809-w.

## Introduction

Accurate detection of ectasia susceptibility is essential during preoperative evaluation for laser vision correction (LVC) surgery because it carries a potential risk of progressive corneal ectasia and subsequent decline in vision after surgery^1–3^. Screening plays a vital role by aiming to identify both subtle forms of ectasia (e.g., subclinical disease) or eyes at potential risk for ectatic change after refractive surgery.

Undiagnosed or subclinical ectatic conditions can lead to symptomatic disease states and adverse visual outcomes if ablative or other tissue altering refractive surgical procedures are performed^4,5^. Additionally, early identification of ectatic disease may allow early intervention to slow or stabilize the disease progression before visual loss^6^. The intricacies in diagnosing mild or subclinical ectatic corneal diseases pose significant challenges. The complexity amplifies the necessity for precise, sensitive, and specific diagnostic modalities. Advanced diagnostic tools are invaluable in appraising the risk of ectasia following LVC and in identifying early disease during the asymptomatic stage^4,5^.

With the advent of tomographic imaging and the multitude of screening programs, the incidence of post-refractive ectasia has decreased, and our ability to diagnose early ectatic disease has substantially improved^1,7,8^. Despite thorough preoperative screening, some cases still evade detection emphasizing the necessity for continued improvement in our diagnostic parameters^9–14^.

The Belin/Ambrósio Enhanced Ectasia Display (BAD) is one of the most commonly used pre-operative refractive screening and ectasia screening progams^15–17^. First introduced in 2008 the original BAD (version 1) displayed anterior and posterior elevation data against a standard best-fit-sphere (BFS) and a newly developed reference surface (enhanced reference surface) in addition to displaying a corneal thickness map and pachymetric progression graphs. The original version did not incorporate any automated machine analysis. Version 2, released in 2010 used a regression analsysis of five tomographic parameters and introduced the final “D” which represented an overall risk analysis. Version 3 (2012 ) added 4 additional parameters to the prior regression analysis with an improved accuracy.

This study uses Scheimpflug tomography indices and sophisticated statistical techniques to construct a more advanced version of the BAD. Version 4 introduces a refined AI with a logistic regression algorithm, trained on a larger dataset, which enhances its diagnostic accuracy, while maintaining the specificity of the display.

## Methods

A multicenter retrospective study was conducted to comply with the revised 2013 Declaration of Helsinki. This study obtained ethical approval from the Institutional Review Board (IRB) and Human Ethics Committee of the Universidade Federal de São Paulo (UNIFESP, SP, Brazil).

The dataset used in this study, was also part of a previously published study^18^, included fully anonymized data of 3,351 patients, totaling 3,886 eyes with no previous surgical history. The data was gathered from 26 international centers, informed consent was obtained from all participants at the time of the data collection, and categorized into four groups. The ‘Normal’ (N) group consisted of one eye randomly selected from 1,665 patients with healthy corneas. The Keratoconus (KC) group was composed of one eye randomly selected from 1,177 patients with clinically diagnosed KC. Additionally, 509 patients were identified with Very Asymmetric Ectasia (VAE) and further subdivided into two subgroups: VAE-E, consisting of 439 unoperated ectatic eyes with clinical ectatic disease disease, and VAE-NT, comprised of 509 eyes from patients with normal topography.

To ensure accurate and reliable data collection, all patients underwent a comprehensive ophthalmic examination, including imaging with the Pentacam HR (Oculus Optikgeräte GmbH; Wetzlar, Germany). Multiple scans were performed for each eye, and only those with a quality specification (QS) score of ‘OK’ were included in the analysis. Patients were instructed to abstain from using soft contact lenses for at least one week before the examination and rigid or hybrid contact lenses for three weeks.

A fellowship-trained expert in corneal and refractive surgery (RA) reassessed all cases’ topographic and tomographic data. The grouping criteria have been previously described elsewhere^19^ and are included in table A (supplemental material). Briefly, the study sample has been divided into four groups: the normal group, which exhibited normal clinical examination and topography; the KC group, which had a diagnosis of ectasia in both eyes; and the very asymmetric ectasia (VAE) group, which included cases with one eye exhibiting a normal clinical and topographic examination (VAE-NT) and the fellow eye diagnosed with ectasia (VAE-E). Only unoperated eyes were included in the study groups.

To ensure the quality of examinations, trained technicians performed the Pentacam HR measurements according to established protocols. For each exam, Scheimpflug photos were manually analyzed by an independent examiner. Using the manufacturer’s software, the Pentacam HR data were transferred to a custom spreadsheet. For each patient, 340 parameters were recorded in an anonymous database, including information from the rotating Scheimpflug tomography and their age at the time of examination.

Statistical computing has been performed using R Core Team (2019) provided by R Foundation for Statistical Computing (Reference: https://www.Rproject.org/), Vienna, Austria. In the analysis, one eye per patient was randomly selected using the dplyr package version 1.1.2 in R. Pre-processing the dataset with 340 attributes included eliminating irrelevant and under-varied features. As a result, 120 attributes were gathered in the final dataset. The dataset was then divided into two separate subsets, with 80% of the data used for training and 20% for testing. To avoid overfitting, variables highly correlated within the training sample were excluded. Shapiro-Wilk test was used to determine if the data followed a normal distribution. Since the parameters in the KC group did not exhibit a normal distribution, the data was presented using the median and interquartile range (IQR). The nonparametric Kruskal-Wallis test was used to compare the parameters across groups. The Dunns’ post hoc test was then applied to facilitate comparisons between each pair of groups. Statistical significance was determined with a p-value below 0.05.

Receiving operating characteristic curves (ROC) were used to assess the differentiating power of each parameter. There were three separate analyses conducted: a comparison between normal and “disease” states (KC + VAE-E + VAE-NT), a comparison between N and clinical ectasia (KC + VAE-E), and a comparison between N and VAE-NT. Based on the Area under the ROC curve (AUROC) of each parameter evaluated, a cutoff value that delivers the highest precision, including sensitivity and specificity metrics, was identified. To assess the performance of diagnostic tests, pairwise comparisons of the AUROC were performed using a nonparametric approach outlined by DeLong et al.^20^.

A logistic regression analysis investigated the relationship between the binary dependant variable Y (normal group against ‘disease’) and potential predictor variables. In the forward Wald optimization, score and Wald statistics significance were used to select predictors, with a p-value threshold of 0.05 for both entry and removal. The likelihood ratio test, the Hosmer-Lemeshow goodness-of-fit test, and the AUROC were all used to evaluate the model’s goodness-of-fit. The final model included 11 variables described in Table 1. The model’s linear term was formulated as follows:

**Table 1:** Description of variables employed in the model.

Variable	Description
age	Age of the patient at the time of examination.
Astig	Measure of the corneal front surface’s astigmatism.
BADDt	Measurement of standard deviations from the mean of the minimum corneal thickness.
BADDy	Measurement of standard deviations from the mean vertical displacement of the minimum corneal thickness.
BADDe	Measurement of standard deviations from the mean of the corneal back elevation.
RPIMax	Maximum pachymetric progression.
ARTmax	Maximum Ambrosio’s Relational Thickness.
RMS HOA (CB)	Root Mean Square of the higher-order aberrations of the corneal back surface.
RMS Z3-Z6 (CF)	Root Mean Square of the Zernike modes of the corneal front surface from the 3rd order up to the 6th order, not including Z(4,0) and Z(6,0).
RMS Z3-Z6 (CB)	Root Mean Square of the Zernike modes of the corneal back surface from the 3rd order up to the 6th order, not including Z(4,0) and Z(6,0).
Z(3,-1)	Measurement of total corneal vertical coma.

*BADD* Standard deviations obtained from the Belin/Ambrósio Enhanced Ectasia Display.

1 $$\:z=\:{\beta\:}_{0}+\:{\beta\:}_{1}*\:{V}_{1}+\:+\:{\beta\:}_{2}*\:{V}_{2}+\dots\:\:+\:{\beta\:}_{11}*\:{V}_{11}$$

In this equation, 'z' represents the linear term of the logistic regression model, ‘z’ is the constant, and.'$\:{\beta\:}_{1}$' to '$\:{\beta\:}_{11}$' are the logistic regression coefficients. Variables '$\:{V}_{1}$' through '$\:{V}_{11}$' are the 11 incorporated variables as outlined in Table 1. To ensure compatibility with the range of the previous version, BAD-D v3, a linear transformation was performed between the logistic model’s linear term 'z' and the scores from BAD-D v3. Using a linear regression analysis, the transformed model outputs were compared to the original BAD-D v3 scores.

## Results

The median age and IQR of participants were 30.5 (16.7) years in the Normal group, 29.6 (13.6) years in the KC group, 27.9 (15.4) years in the VAE-NT group, and 28.2 (15.3) in the VAE-E group. Even though mild, the differences in age among groups were statistically significant (*p* < 0.001); in pairwise post hoc Dunn’s test comparisons, only the normal group was statistically different from the other three, whereas the others were not statistically different. The right: left eye ratio did not differ significantly among the groups (*p* = 0.999).

The distribution of some tomographic indices comprising the anterior (corneal astigmatism and maximum anterior axial curvature [KMax]) and posterior (maximum posterior elevation concerning the best fit toric ellipsoid) surfaces along with the minimum corneal thickness among the groups are described in Table 2. The four groups had statistically significant differences for all BBvariables (*p* < 0.001). In pairwise comparisons, it was found that the difference between the Normal and VAE-NT was not significant with KMax (*p* = 0.328) and that most of the indices between the KC and VAE-E were not significantly different (*p* > 0.587), except for the corneal thickness minimum (*p* = 0.001).

**Table 2:** Distribution of corneal Tomographic Indices among groups.

	Normal		KC		VAE-NT		VAE-E			Pairwise group comparisons (*p*-value)					
	median	iqr	median	iqr	median	iqr	median	iqr	KW (p-value)	N vs. KC	N vs. VAE-NT	KC vs. VAE-NT	N vs. VAE-E	KC vs. VAE-E	VAE-NT vs. VAE-E
Astig (D)	1.0	0.9	2.8	2.6	0.9	0.8	3	2.5	< 0.001	< 0.001	0.005	< 0.001	< 0.001	0.587	< 0.001
KMaxFront (D)	44.5	2.0	52.3	7.8	44.7	2.2	51.6	7.6	< 0.001	< 0.001	0.328	< 0.001	< 0.001	1.000	< 0.001
EleBBFTE8mmMax (µm)	6	2	37	29	7	3	36	29.5	< 0.001	< 0.001	< 0.001	< 0.001	< 0.001	1.000	< 0.001
PachyMin (µm)	547	41	471	53	519	39	486	49.5	< 0.001	< 0.001	< 0.001	< 0.001	< 0.001	0.001	< 0.001

*Astig* corneal front surface’s astigmatism,* KMaxFront* Maximum corneal front surface’s axial curvature,* EleBBFTE8mmMax* Maximum corneal back surface’s elevation in relation to best fit toric ellipsoid,* PachyMin* Minimum corneal thickness,* iqr* interquartile range,* KW* Kruskal-Wallis test,* N* Normal group,* KC* Keratoconus group,* VAE-NT* Normal topography eye of very asymmetric ectasia cases,* VAE-E* Ectatic eye of very asymmetric ectasia cases.

**Fig. 1:**
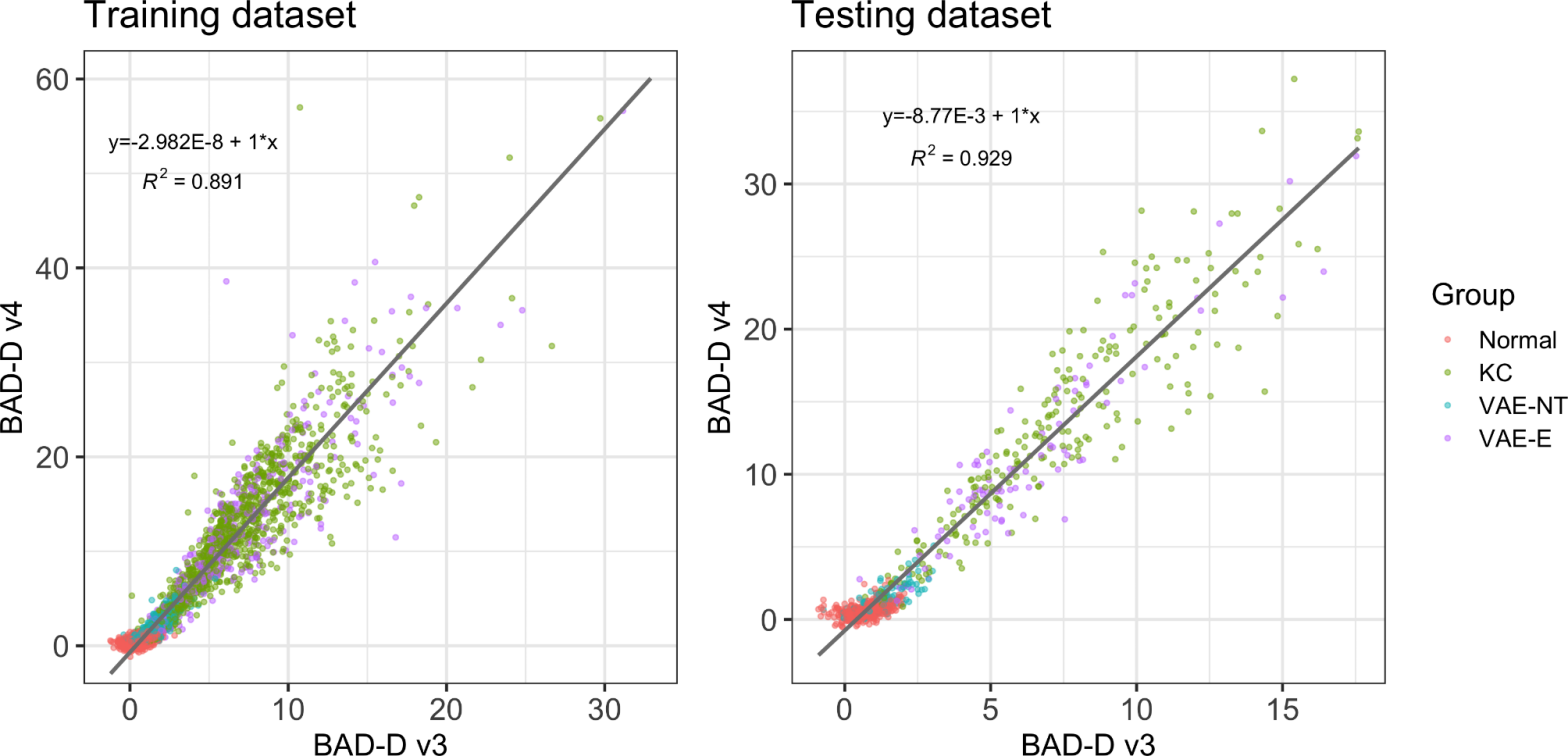
Linear relationship between BAD-D v3 and BAD-D v4 in training and testing samples.

Table 3 shows the distribution of the 11 predictors selected for the final model in the training and test samples. Variable distributions between Training and Testing samples were well-balanced, with no statistically significant differences. Table 4, 5, 6 summarizes the accuracy of these predictors in discriminating between Normal and Clinical Ectasia (KC + VAE-E), Normal and “Disease” (KC + VAE-E + VAE-NT), and Normal and VAE-NT. Figure 1 outlines the linear relationship between the BAD-D v3 and BAD-D v4 in training and testing samples.

**Table 3:** Distribution of the Model’s variables in the training and test samples.

	Normal			KC			VAE-NT			VAE-E		
	Train	Test	p-value	Train	Test	p-value	Train	Test	p-value	Train	Test	p-value
Age (years)	30.41 (16.8)	30.83 (16.24)	0.751	29.86 (13.42)	29.2 (13.94)	0.109	28.12 (15.44)	27.66 (16.5)	0.791	28.36 (14.94)	26.59 (15.84)	0.372
Astig (D)	1 (0.9)	1 (0.9)	0.744	2.8 (2.6)	2.8 (2.5)	0.755	0.9 (0.8)	0.8 (0.7)	0.710	3 (2.5)	3.2 (3)	0.679
BADDt	-0.27 (1.12)	-0.24 (1.1)	0.730	2.16 (1.94)	2.34 (1.98)	0.392	0.62 (1.23)	0.45 (1.19)	0.325	1.6 (1.79)	1.71 (1.66)	0.913
BADDy	0.43 (1.12)	0.4 (1.18)	0.317	0.95 (1.4)	0.95 (1.33)	0.794	0.98 (1.14)	0.94 (1.14)	0.295	0.93 (1.54)	1.1 (1.19)	0.584
BADDe	0.02 (0.91)	0.12 (0.92)	0.148	7.67 (6.4)	8.24 (6.24)	0.457	0.81 (1.26)	0.63 (1.27)	0.103	7.67 (6.39)	6.91 (4.66)	0.143
RPIMax	1.18 (0.23)	1.17 (0.2)	0.856	2.63 (1.09)	2.71 (1.27)	0.242	1.4 (0.36)	1.37 (0.28)	0.290	2.57 (1.23)	2.46 (0.71)	0.291
ARTmax	466 (103)	470 (91)	0.957	178.5 (88)	174 (88)	0.257	368 (110.5)	371 (92.75)	0.275	189.5 (108.25)	199 (72.5)	0.352
RMS HOA (CB)	0.19 (0.04)	0.18 (0.04)	0.498	0.62 (0.44)	0.65 (0.44)	0.176	0.21 (0.07)	0.2 (0.06)	0.207	0.6 (0.44)	0.56 (0.33)	0.335
RMS Z3-Z6 (CF)	0.06 (0.03)	0.06 (0.03)	0.460	0.46 (0.4)	0.48 (0.39)	0.284	0.08 (0.04)	0.07 (0.03)	0.079	0.42 (0.39)	0.38 (0.34)	0.542
RMS Z3-Z6 (CB)	0.02 (0.01)	0.02 (0.01)	0.269	0.13 (0.1)	0.14 (0.1)	0.139	0.03 (0.02)	0.03 (0.01)	0.074	0.13 (0.09)	0.12 (0.07)	0.488
Z(3,-1)	0 (0.17)	0.01 (0.16)	0.625	-0.02 (0.65)	0.02 (0.68)	0.134	0 (0.21)	0 (0.18)	0.799	0.01 (0.77)	0.04 (0.74)	0.759

*KC* Keratoconus group,* VAE-NT* Normal topography eye of very asymmetric ectasia cases,* VAE-E* Ectatic eye of very asymmetric ectasia cases,* Age* Age of the patient at the time of examination,* Astig* Measure of the corneal front surface’s astigmatism,* BADDt* Measurement of standard deviations from the mean of the minimum corneal thickness,* BADDy* Measurement of standard deviations from the mean vertical displacement of the minimum corneal thickness,* BADDe* Measurement of standard deviations from the mean of the corneal back elevation,* RPIMax* Maximum pachymetric progression,* ARTmax* maximum Ambrosio’s relational thickness,* RMS HOA (CB)* root mean square of the higher-order aberrations of the corneal back surface,* RMS Z3-Z6 (CF)* root mean square of the Zernike modes of the corneal front surface from the 3rd order up to the 6th order, not including Z(4,0) and Z(6,0),* RMS Z3-Z6 (CB)* root mean square of the Zernike modes of the corneal back surface from the 3rd order up to the 6th order, not including Z(4,0) and Z(6,0); Z(3,-1): Measurement of total corneal vertical coma.

**Table 4:** A accuracy of predictors in discriminating between normal and clinical Ectasia (KC + VAE-E).

	Training				Testing			
	AUC	Sensitivity	Specificity	Threshold	AUC	Sensitivity	Specificity	Threshold
Age (years)	0.5428	0.8224	0.2528	40.97	0.5832	0.2226	0.9279	21.16
Astig (D)	0.8352	0.673	0.8657	2.05	0.8245	0.6774	0.8799	2.05
BADDt	0.9381	0.8277	0.9047	0.84	0.9376	0.8129	0.9489	1
BADDy	0.6472	0.4786	0.7532	1	0.6721	0.6097	0.6577	0.74
BADDe	0.9821	0.9311	0.982	1.86	0.9842	0.9161	0.994	1.9
RPIMax	0.9855	0.9342	0.9797	1.54	0.9812	0.9161	0.982	1.56
ARTmax	0.9888	0.9456	0.9775	342.5	0.9845	0.9161	1	320.5
RMS HOA (CB)	0.9743	0.9196	0.967	0.26	0.9666	0.9161	0.979	0.27
RMS Z3-Z6 (CF)	0.9794	0.9395	0.9715	0.04	0.9773	0.929	0.985	0.05
RMS Z3-Z6 (CB)	0.9921	0.9364	0.9827	0.12	0.9924	0.9323	0.988	0.13
Z(3,-1)	0.5069	0.3147	0.9625	-0.22	0.5232	0.3484	0.979	0.26

**Table 5:** Accuracy of predictors in discriminating between normal and “Disease” groups (KC + VAE-E + VAE-NT).

	Training				Testing			
	AUC	Sensitivity	Specificity	Threshold	AUC	Sensitivity	Specificity	Threshold
Age (years)	0.5512	0.1809	0.907	21.06	0.5814	0.22	0.9309	21.08
Astig (D)	0.7395	0.5304	0.8657	2.05	0.7368	0.545	0.8799	2.05
BADDt	0.896	0.7386	0.8942	0.78	0.8939	0.7425	0.9099	0.82
BADDy	0.6548	0.4771	0.7584	1.06	0.6709	0.5975	0.6577	0.74
BADDe	0.924	0.7878	0.9482	1.34	0.9131	0.74	0.994	1.88
RPIMax	0.9372	0.8151	0.955	1.46	0.9349	0.7925	0.961	1.5
ARTmax	0.947	0.8406	0.9565	359.5	0.9437	0.7775	0.988	333
RMS HOA (CB)	0.8995	0.7641	0.9497	0.25	0.8923	0.7525	0.97	0.26
RMS Z3-Z6 (CF)	0.9277	0.7849	0.967	0.04	0.9191	0.8275	0.9189	0.03
RMS Z3-Z6 (CB)	0.925	0.8133	0.9295	0.1	0.9226	0.7925	0.952	0.1
Z(3,-1)	0.5037	0.2504	0.9745	-0.24	0.5153	0.28	0.979	0.26

**Table 6:** Accuracy of predictors in discriminating between normal and “Disease” groups (KC + VAE-E + VAE-NT).

	Training				Testing			
	AUC	Sensitivity	Specificity	Threshold	AUC	Sensitivity	Specificity	Threshold
Age (years)	0.5512	0.1809	0.907	21.06	0.5814	0.22	0.9309	21.08
Astig (D)	0.7395	0.5304	0.8657	2.05	0.7368	0.545	0.8799	2.05
BADDt	0.896	0.7386	0.8942	0.78	0.8939	0.7425	0.9099	0.82
BADDy	0.6548	0.4771	0.7584	1.06	0.6709	0.5975	0.6577	0.74
BADDe	0.924	0.7878	0.9482	1.34	0.9131	0.74	0.994	1.88
RPIMax	0.9372	0.8151	0.955	1.46	0.9349	0.7925	0.961	1.5
ARTmax	0.947	0.8406	0.9565	359.5	0.9437	0.7775	0.988	333
RMS HOA (CB)	0.8995	0.7641	0.9497	0.25	0.8923	0.7525	0.97	0.26
RMS Z3-Z6 (CF)	0.9277	0.7849	0.967	0.04	0.9191	0.8275	0.9189	0.03
RMS Z3-Z6 (CB)	0.925	0.8133	0.9295	0.1	0.9226	0.7925	0.952	0.1
Z(3,-1)	0.5037	0.2504	0.9745	-0.24	0.5153	0.28	0.979	0.26

The BAD-D v4 significantly improved the ability to distinguish between ‘Normal’ and ‘Clinical Ectasia’ in its most recent iteration. An AUROC of 0.997 (95% CI, 0.995–0.998) was associated with 97.81% sensitivity and 99.17% specificity at the 1.80 cutoff value for the training sample. Similarly, the testing sample recorded an AUROC of 0.998 (95% CI, 0.996–0.999) along with 98.06% sensitivity and 98.80% specificity at the 1.89 cutoff value. There was no statistically significant difference between these two samples (*p* = 0.472), attesting to the consistency of BAD-D v4. The previous version of the index, BAD-D v3, had an AUROC of 0.994 (95% CI, 0.991–0.997), a sensitivity of 96.3%, and a specificity of 99.0% at a cutoff of 1.87. Compared with BAD-D v4, these results were statistically less accurate (*p* < 0.05).

In distinguishing between ‘Normal’ and ‘Disease’, the BAD-D v4 train training and testing ining sample’s AUROC was 0.974 (95% CI, 0.969–0.979) at the 1.47 cutoff value, with a sensitivity of 89.97% and specificity of 96.70%. The testing sample paralleled these results with an AUROC of 0.966 (95% CI, 0.953–0.979), 88.75% sensitivity, and 96.40% specificity at the 1.45 cutoff. The statistical similarity of these findings (*p* = 0.229) underscores the consistency of BAD-D v4’s performance. However, when we examine the performance of the preceding iteration, BAD-D v3, it presents an AUROC of 0.952 (95% CI, 0.945–0.958) with a sensitivity and specificity of 86.26% and 94.72%, respectively, for the 1.52 cutoff value. There was a statistically significant decrease in accuracy compared with BAD-D v4 in both the training and testing samples (*p* < 0.05).

As for the differentiation between ‘Normal’ and ‘VAE-NT’, The BAD-D v4 achieved an AUROC of 0.905 (95% CI, 0.888–0.922) with a sensitivity of 79.47% and a specificity of 86.42% at the 1.07 cutoff value in the training sample, similarly, the testing sample yielded an AUROC of 0.858 (95% CI, 0.809–0.906), with 73.33% sensitivity and 88.59% specificity. These results’ statistical non-significance (p = 0.074) affirms BAD-D v4’s consistent performance across different scenarios. The prior version, BAD-D v3, exhibited an AUROC of 0.789 (95% CI, 0.732–0.847) with a sensitivity of 67.78% and a specificity of 81.99%, at the 1.22 cutoff value. The decrease in performance relative to BAD-D v4 was statistically significant in training and testing samples (p < 0.05).

**Fig. 2:**
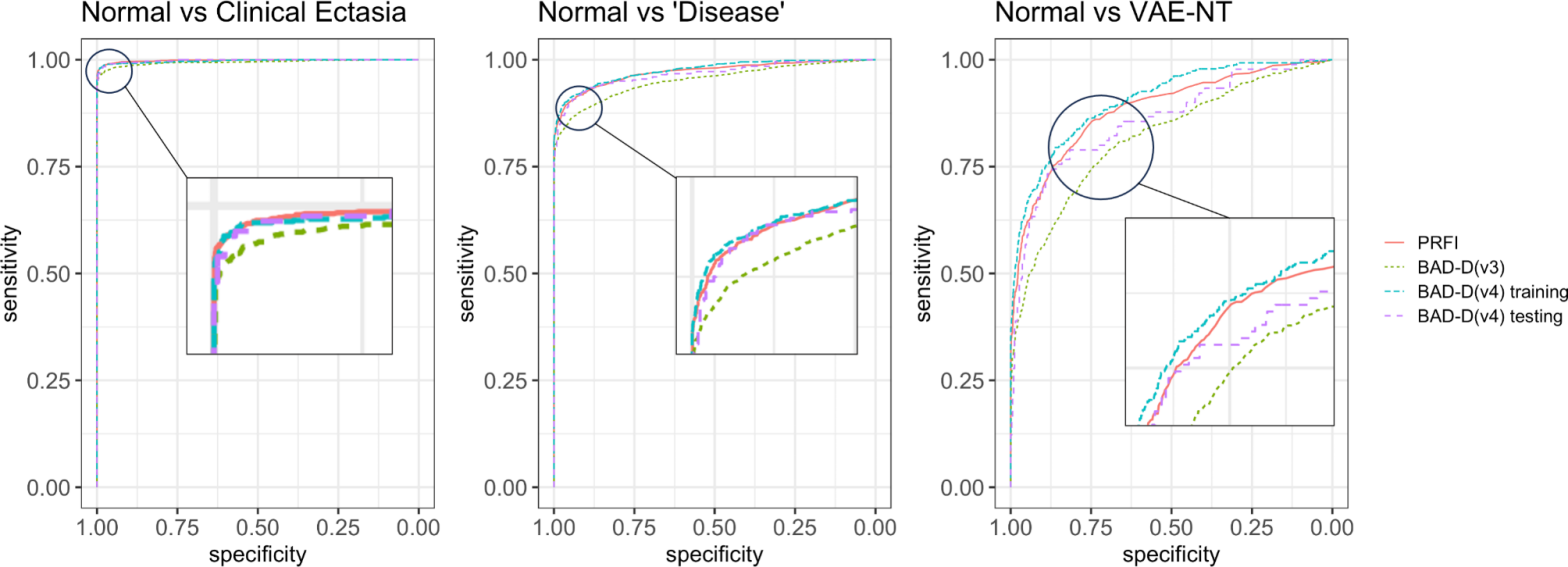
ROC curve analysis comparing BAD-D (v3), BAD-D (v4), and PRFI in differentiating normal from clinical ectasia (KC + VAE-E), Normal from “Disease” (KC + VAE-E + VAE-NT), and Normal from VAE-NT.

Figure 2 illustrates these results.

In all groups, the PRFI’s performance significantly differed from BAD-D v3 (*p* < 0.001), but showed no statistical difference when compared with both the training and testing samples of BAD-D v4 (*p* > 0.05), as shown in Fig. 2.

## Discussion

In this study, a novel index anchored exclusively on corneal tomography data was constructed. As opposed to the previously published and more complex Pentacam Random Forest Index (PRFI)^21^, this study used the simpler logistic regression analysis with similar resuls in both sensitivity and specificity. The added benefit to the logistic regression method it that it can be incorporated into the current Belin/Ambrósio Enhanced Ectasia Display (BAD) without altering the end-user experience as most refractive surgeons and corneal specialist are already familiar with the clinical application of the display. The findings revealed that BAD-D v4 is exceptionally efficient in differentiating heatlhy corneas from frank ectasia, and also performs well in diagnosing milder/susceptible forms such as the VAE-NT. It consistently outperformed BAD-D v3 and matched the PRFI accuracy.

The efficacy of our updated model in enhancing the accuracy of ectasia detection underscores the vital role of tomographic data. Nevertheless, a critical evaluation of the results prompts us to consider whether we may be nearing the limits of the potential that tomographic data can provide in this context. For instance, our results in distinguishing ‘Normal’ from ‘VAE-NT’ reported an AUROC of 0.905 for the training sample and 0.858 for the testing sample using BAD-D v4. This was the same cap (AUROC around 0.9) of previous models using different tomographic devices^11–14,22,23^. This result could hint at the inherent limitations of relying solely on tomographic data, suggesting that even with the most advanced models, some aspects of ectasia might remain challenging to detect exclusively with this type of data.

Data on 120 attributes were initially collected; however, the logistic regression analysis used only 11 parameters. This selection was made because the inclusion of additional parameters was found not to enhance the AUROC. Although this held true for the parameters tested, it is suggested that future investigations consider non-tomographic derived data, such as biomechanics, to determine if such data can elevate the overall AUROC^18,24,25^.

Posterior elevation has a well-recognized role in the detection of early keratoconus^26–29^. In our study, the maximum corneal back surface elevation relative to the best-fit toric ellipsoid was significantly different between the normal group and each of the three ectasia groups (*p* < 0.001 for all pairwise comparisons). Additionally, the VAE-NT group showed significant differences compared to both the VAE-E and KC groups (*p* < 0.001), whereas no statistically significant difference was observed between the VAE-E and KC groups (*p* > 0.05).

There are some limitations inherent to the present study. One was the use of VAE-NT cases. Even though these cases are routinely used in clinical settings to simulate real-world conditions, they may, in some cases, represent true unilateral ectasia^30^. Keratoconus is traditionally described as an asymmetric disease, differences between the eyes may aid in its challenging early detection^31,32^. The inclusion of an extremely asymmetric group by definition in this study may bias the analysis of inter-eye asymmetry. Future studies designed to exclude this group can better evaluate the diagnostic potential of inter-eye asymmetry. While we have compared BAD-D v4 with v3 and the PRFI, we acknowledge that direct comparisons with other AI algorithms are limited due to lack of access to their proprietary algorithms and datasets. Future studies could address this limitation by exploring more comprehensive comparisons when such data become available. Ideally, the normal group should include preoperative scans from LVC cases that did not develop ectasia within the first three years. However, this group was not available in our dataset and, therefore, was not included in this study. Future studies should aim to include such cases to better assess the long-term ectasia risk in post-surgical populations. Moreover, the dataset’s diverse demographic and ethnic composition may require future studies to control for these variables to better understand their influence. Although intraocular pressure (IOP) normalization is not routine in clinical practice and was not explicitly performed in this study, we acknowledge that IOP can influence tomographic measurements. However, as normalization of IOP is not a routine part of LVC screening, the clinical value of prior normalization is probably limited.

An additional limitation is the exclusion of less common “natural” ectasia phenotypes, including pellucid marginal degeneration, keratoglobus, and other corneal diseases, such as Fuchs endothelial dystrophy, and postoperative stable cases after laser vision correction. There is a possibility that the findings may be limited in scope if these cases are absent, as they generally have characteristic features that facilitate clinical diagnosis. Therefore, future studies should evaluate whether advanced indices, such as the novel BAD-D v4, can be used to identify these diverse corneal diseases.

## Conclusions

In conclusion, our study establishes the substantial efficacy of the novel BAD-D v4 model, which is grounded exclusively on the analysis of corneal tomography data using logistic regression analysis. The model demonstrated comparable, if not superior, performance to previous versions and even other sophisticated indices like the PRFI through various group comparisons. The unique advantage of aligning with the current BAD-D display is that it streamlines the user experience, facilitating an easy transition for surgeons already familiar with the existing framework.

## Electronic supplementary material

Below is the link to the electronic supplementary material.

Supplementary Material 1

## Data Availability

The datasets generated and/or analyzed during the current study are not publicly accessible but are available from the corresponding author on reasonable request.
