# Supplementary material for: Enhancing corneal ectasia susceptibility detection: analysis of a new algorithm (BAD-D v4)
Source: Sci Rep. 2024 Dec 4;14:30226. doi: 10.1038/s41598-024-81809-w (PMC11618354; doi:10.1038/s41598-024-81809-w)
Supplement: Supplementary file 1 — Supplementary Material 1 [file 41598_2024_81809_MOESM1_ESM.docx]

Table A: Grouping criteria for study participants.

| Group | Inclusion criteria |
| --- | --- |
| Normal | Normal corneas on general eye examination in both eyes, including normal slit lamp biomicroscopy.  Corrected distance visual acuity of 20/20 or better.  Overall subjective normal topography and tomography examinations.  No previous ocular surgery. |
| Keratoconus | Clinical ectasia diagnosed in both eyes.  **Topographic Characteristics:** Presence of skewed asymmetric bowtie pattern or inferior steepening.  **Slit-Lamp Findings:** At least one sign such as Munson's sign, Vogt's striae, Fleischer's ring, apical thinning, or Rizutti's sign.  No previous ocular procedures (e.g., corneal cross-linking [CXL], intracorneal ring segments implantation). |
| Very asymmetric Ectasia – Ectasia Eye | Clinical ectasia confirmed in one eye based on the criteria used for the Keratoconus group.  **Fellow Eye:** The fellow eye has a normal front surface curvature (topometric) map as described below. |
| Very asymmetric Ectasia – Normal Topography eye | **Topographic Characteristics:** Front surface objective curvature metrics derived from Pentacam, including:   - Keratoconus percentage index (KISA%) score lower than 60.   - Paracentral inferior–superior (I-S) asymmetry value at 6 mm (3-mm radii) less than 1.45.  These objective standards were chosen to prevent issues with subjectivity and variability in topographic map classifications between and among examiners. |
